# Structure and Functions of the Lymphatics

**Published:** 1868-07

**Authors:** 


### ARTICLE II.

### *Structure and Functions of the Lymphatics.*

Much has been recently written on the question of the structure and functions of the lymphatic system, and old notions have been considerably modified. A brief summary of the latest results will, we hope, not be unacceptable to the readers of the Journal.

It can hardly be necessary to say that the lacteals form a part of the lymphatic system and that any general study of the one applies equally to the other. The proper order of inquiry is first to examine the mode of origin of the lymphatics and lacteals; secondly, to study the structure of the lymphatic glands and of those structures so nearly related to them, Peyer's glands; and thirdly, to follow the lymph in its passage through these organs.

First, as to the origin the lymphatics and lacteals; are they continuous with the blood-vessels through a very delicate system of capillary vessels, (the *vasa serosa* of the older writers) admitting only liquor sanguinis without corpuscles; or do they begin by an independent anastomotic plexus; or do they originate in the passages of areolar tissue; or finally do they arise from the hollow corpucles and caudate cells, of the this tissue? Now, as to those fine vessels which only became visible during inflammation, we know they are continuous not with the lymphatics but the veins. Teichmann declares that the lymphatics first originate in stellate cells, the elongated processes of which forms an irregular network, and then coalesce with large tubes which gradually acquire valves. Kolliker seems to corroborate these observation in some particulars, but inclines to the opinion that they arise from closed free extremities.

The difficulty of admitting the existence of an origin by closed capillary tubes parallel with the blood-vessels is a physical one. It is easy to conceive how by the *vis a tergo* of the heart, the lymph may be forced out though the blood-vessels into the areolar inter-spaces, but how can it get into the lymphatics, if they are closed on all sides? Why should it not travel along the less resisting and looser meshes of the areolar tissue outside of the vessels?

The more recent observations appear to establish the fact, that among these very inter-spaces the lymphatic system originates. The cavities of the common areolar tissue seem first to form splits and irregular spaces, which gradually close up into channels without proper walls, and are finally, by the condensation of tissue, formed into regular vessels. The lymph feeling the impulse of the heart, courses through these channels and finally taking advantage of the valves, moves along in a current of considerable rapidity. Sometimes the change from mere splits into vessels is sudden and abrupt, at others more gradual through the medium of imperfectly formed vessels. Then the lymphatics seem essentially to belong to the connective tissue, forming confluent lacunæ which are continuous with vessels, the walls of which become more and more consolidated and at last lined with an epithelium.

The question of the origin of the lacteals and the mode in which the chyle enters them is still more obscure than that problem of which we have just been treating. We all know that the cells of the intestinal villi will take an active part in the absorption of the chyle, but what that part is, is not quite so clear. These cells are described as cylindrical, containing only fine granules and an oval, vesicular nucleus with one or two nucleoli. Water separates the cell contents from the broad end, producing a clear border or seam in separate cells, and in series of cells a coat like that of the structureless cuticle of plants. Some observers insisted that these epithelial cells were open at both their free and attached extremities, constituting funnels which admitted the entrance of fat. Much discussion has taken place upon this question, in which the views just mentioned have been attacked, defended and modified, but no definite conclusion has been reached.

However originating, we know that these vessels combine into large trunks and pass through the lymphatic glands. Concerning these structures, discussion has been as active as upon other portions of the lymphatic system. Without alluding to the old views of the structure of these glands entertained by Hewson, Malpighi, Morgagni and others, we pass at once to the discoveries of the modern microscopists, Billroth, His, Frey and Teichmann.

The simplest form of these glands, is that of a conformed plexus of lymphatics and blood-vessels. The former divide and subdivide very minutely and form occasional anastomoses, retracting into a trunk of exit. They are destitute of valves and rarely convoluted. Among these fine lymphatics lies a mass of anastomosing capillaries which have no communication with the lymph vessels. All these are bound together by connective tissue, containing elastic fibres, which invests the whole gland and dips down among the structures already described.

His describes the lymphatic glands as forming oval or bean shaped bodies, receiving afferent vessels on their convex surface, and presenting on the surface a hilus from which the afferent vessels and the veins emerge, and into which the larger arterial trunks enter. In this hilus can be detected not only the connective tissue, but also two distinct glandular structures, the cortical and the medullary. The former is of a lighter colour and of a granular texture, the minute grains being only partially separated from each other by processes of cellular tissue dipping in from the general investing sheath. They are of a yellowish or reddish-yellow colour, large and better defined near the surface, becoming smaller, less separated from each other and more angular towards the medullary substance with which they ultimately blend. The medullary tissue is soft and spongy, reddish, brown or black, containing a few large blood-vessels, and traversed by numerous fine passages, serving as conduits for lymph and chyle.

In considering these different structures, it is better to examine them separately. The *stroma of the hilus* consists of connecting tissue, with fat-cells, blood-vessels and lymph-vessels. It is whitish, tough and apparently porous. It is most abundant in those glands in which there is the least true medullary substance for which it has often been mistaken.

The *general investing sheath* is composed of connective tissue with numerous spindle shaped corpucles interspersed among the fibrils. Externally it is loose and continuous with the common fascia, while internally, it gives off processes or septa dipping into the substance of the gland and constituting trabecular tissue. In some glands muscular fibres are distributed among these septa, while in others they are absent. The arrangement of the trabeculæ differ in the cortical and in the medullary tissue, in the former enclosing circular spaces and in the latter, forming a kind of trellis work with close meshes and delicate fibres.

If no other structure were present the trabeculæ would form a series of communicating cavities, ampulla-form and large near the surface, but constituting a sort of branched canal system in the medullary substance. This whole system of cavities may be injected from the afferent vessels, if sufficient pressure is employed.

The *medullary substance* is composed of a network of connective tissue full of blood vessels and containing a large number of lymph corpuscles in its interstices. In the vertical part, it occupies the cavities formed by the trabeculæ of the sheath but does not completely fill them, a space being left between the surfaces of the trabeculæ and of the medullary substance, which is traversed by a few coarse fibres holding the two together. This union has been compared to the canvass of worsted work held in its frame by threads. The space is the lymph sinus and varies from 1-2500 to 1-800 of an inch in diameter. Towards the centre of the gland the medullary substance forms a well defined mass, but the lymph sinuses exist here too, but assume the form of tubes, and are continuous with the afferent and efferent vessels. Deep in this medullary substance are found vacant spaces called vacuolæ. Some of the arteries follow the trabeculæ while others occupy the axis of the medullary canals. In these last they send branches towards the periphery which ultimately form an anastomotic, capillary plexus surrounding the canals. From this plexus spring the veins and their trunks also occupy the axis of the canals. The follicles are supplied from the trunks of the medullary substance, never from those of the trabeculæ.

The connective tissue forms a delicate cellular network with variously formed meshes having usually caudate prolongations. After food, especially if it has been oily, these cells and their prolongations contain fat-globules, giving them an opaque and milky appearance. The lymph tubes have but a single transparent, colorless membrane, and contain lymph corpuscles and during digestion fat-globules. The blood vessels traversing their axis have lost their external coat, so that the lymph and blood readily react upon each other by osmosis. We have spoken of these as tubes, because it is under this title that Frey describes them, but it must be remembered that they are only channels formed among the vessels of the connective tissue, and are really cavities in the medullary structure.

The course of the circulation is very complex through all these tortuous channels, connected indeed with the afferent vessels but not formed by them. Retarded movement is the result of the progress through this system of tubes and cavities, so that abundant facilities are afforded for reciprocal action between the lymph and the blood. Lymph corpuscles are slowly formed in the interstices we have endeavored to describe, and the lymph, which is the surplus liquor sanguinis left from the nutritive processes, circulates chiefly in the larger channels on the surface of the gland. After food, the flow of lymph and chyle is greatly increased, the lymph channels in both cortical and medullary are much distended, and afford passage to the lymph corpuscles from the granular tissue, in which they are developed, into the lymph sinuses and thence into the afferent lymphatic vessels.

The question of the point of origin may be still be considered open. It is generally acknowledged that they are formed in the glands, but recent researches show that they may also originate in the minute vessels and stellate cells, in which some observers believe the lymphatic system to originate.

The latest chemical analysis of lymph was made by Henson. The liquid was obtained from the thigh of a patient suffering with Barbadoes leg or a severe analagous affection. It had a whitish turbid appearance; passed quickly into putrefaction, possessed an alkaline reaction and had a specific gravity of 1.007.

1000 parts contained

| | |
|---|---|
| Water | 987.7 |
| Solids | 12.3 |
| | 1000.0 |

| | | |
|---|---|---|
| Fat | | 0.030 |
| Organic extractives soluble in alcohol | | 1.284 |
| Organic substances soluble in water, extractive and albumen | | 0.908 |
| Organic substances insoluble in water, alcohol-fibrin and insoluble albumen | | 1.699 |
| Inorganic substances soluble in water | | 8.076 |
| Chloride of Sodium | 6.148 | |
| Soda | 0.573 | |
| Potash | 0.496 | |
| Carbonic acid | 0.638 | |
| Sulphuric and phosphoric acid and loss | 0.221 | |
| Inorganic substances insoluble in water | | 0.303 |
| Chalk | 0.132 | |
| Magnesia | 0.011 | |
| Oxide of iron | 0.006 | |
| Phosphoric acid | 0.118 | |
| Carbonic acid | 0.015 | |
| Carbonate of magnesia, sulphuric and loss. | 0.021 | |

A more minute examination of the lymph showed that 1000 grains contained of

| | |
|---|---|
| Fibrine imperfectly purified | 1.070 |
| Serum albumen | 1.408 |
| Albuminous compounds precipitated by acetic acid | 0.894 |

